# Dealing with pre-existing chronic neutropenia in cancer patients—considerations and consequences in the clinical praxis

**DOI:** 10.3332/ecancer.2020.1131

**Published:** 2020-10-29

**Authors:** Anna-Birgitte Thinggaard, Gabor Liposits, Niels Fristrup

**Affiliations:** 1 Department of Oncology, Regional Hospital West Jutland, Denmark; 2 Department of Oncology, Aarhus University Hospital, Denmark; a https://orcid.org/0000-0002-8204-3949

**Keywords:** chronic neutropenia, autoimmune neutropenia, chronic idiopathic neutropenia, chemotherapy, targeted therapy, monoclonal antibodies

## Abstract

Chronic neutropenia is a rare but important challenge with substantial clinical implications for patients receiving antineoplastic treatment. Treatment-induced neutropenia is a well-known adverse event during chemotherapy and some targeted treatments. Guidelines for administering chemotherapy are rather strict to protect the patient from severe and life-threatening complications. Consequently, patients with chronic neutropenia may receive suboptimal antineoplastic treatment. Autoimmune neutropenia or chronic idiopathic neutropenia (CIN) may affect the antineoplastic treatment by causing delayed drug delivery, dose reductions and early discontinuation of treatment. CIN is characterised by the onset in late childhood or adulthood, affects mostly women, is clinically benign and has rare spontaneous remission. Here, we elucidate the challenges related to chronic neutropenia when administering chemotherapy through two clinical cases. Guidelines may need to be revised in order to optimise the treatment of patients with asymptomatic chronic neutropenia, thus personalising the medical decisions for each patient.

## Introduction

Neutropenia is characterised by abnormally low levels of neutrophil granulocytes (further neutrophils) in the blood. The neutrophils play an important role in the immune response and neutropenia is known to increase the risk of serious infections [1]. The normal neutrophil count is defined between 2 and 7 × 10^9^/L, with levels below 1.5 × 10^9^/L defined as clinically relevant neutropenia [1]. Neutropenia is a well-known adverse event related to chemotherapy and consequently increases the risk of infection during treatment in patients with cancer.

Neutropenia is divided into three categories depending on the severity of the condition: mild (>1 × 10^9^/L), moderate (0.5–1 × 10^9^/L) and severe neutropenia (<0.5 × 10^9^/L) [2].

Febrile neutropenia is a potential life-threatening adverse event related to systemic antineoplastic treatments (Table 1), leading to hospital admission, and may cause delay in treatment administration, and result in either dose reduction or treatment withdrawal, or addition of granulocyte colony-stimulating factor (G-CSF) [3]. Prior to chemotherapy administration, new haematological tests are required to ensure appropriate bone marrow function. The administration of chemotherapy or other drugs inducing neutropenia requires a certain level of neutrophils and thrombocytes, although it varies among treatment regimens [4]. If these requirements are not met, the treatment is contraindicated and will be postponed until the bone marrow function recovers.

An example of non-chemotherapy drugs inducing neutropenia is cyclin-dependent kinase 4/6 inhibitors (CDK4/6i). These targeted agents lead to cell cycle arrest (G1-S phase) [5]. This in turn leads to senescent-like cellular phenotype in the tumour cells [6]. A common adverse event related to the administration of CDK4/6i is severe neutropenia [5].

The prevalence of neutropenia in Caucasians is estimated being less than 1% and chronic neutropenia approximately 0.1% [7, 8]. Chronic neutropenia is classified as a neutrophil count <1.5 × 10^9^/L that persists for more than 3 months [9]. The condition spans widely; it occurs in all ages and may be insignificant or life-threatening. A range of extrinsic and intrinsic factors may cause chronic neutropenia, e.g., bone marrow dysfunction or malnutrition/starvation [9]. Table 2 summarises the factors or conditions that are a common cause for selective neutropenia, when neutropenia is first diagnosed and proven persistent in both time and multiple blood tests.

If no obvious reason for the neutropenia can be found, a bone marrow biopsy is usually recommended. Only if all tests are normal, ‘idiopathic neutropenia’ can be diagnosed [9, 10].

Chronic idiopathic neutropenia (CIN) and autoimmune neutropenia (AIN) are often interchangeable in adults since no reliable antibody test is yet available [1]. AIN is characterised by antineutrophil antibodies circulating in the blood stream. It can be idiopathic (primary) or secondary. Secondary AIN is caused by some other underlying (autoimmune) disease. CIN is not associated with other pathologies. CIN is characterised by the onset in late childhood or adulthood, affects more women than men, is clinically benign and has rare spontaneous remission, whereas primary AIN is more frequent in children and often self-limiting [9, 11].

Here, we present two cases of chronic neutropenia in colon cancer (case #1) and ovarian cancer (case# 2) and highlight the dilemma of suboptimal anti-neoplastic treatment.

Colorectal cancer (CRC) is the third most common cause of cancer-related deaths in women [12].

Capecitabine (an oral prodrug of 5-fluorouracil) and 5-fluorouracil have long been the backbone of chemotherapy for stages II–III colon cancer in the adjuvant setting [13]. The duration of treatment has been proven most efficient when given for six months [14]. Haematological treatment requirements are neutrophils ≥1.5 × 10^9^/L and thrombocytes ≥100 × 10^9^/L.

Ovarian cancer is the eight most common cause of cancer-related deaths in women [12]. Mortality rates and survival depend greatly on age and are especially poor in women +70-year old [15].

Carboplatin, doxorubicin and paclitaxel are among the most effective cytotoxic agents used for the systemic treatment of ovarian cancer [16]. For carboplatin in combination with doxorubicin, the haematological treatment requirements are neutrophils ≥1.5 × 10^9^/L and thrombocytes ≥100 × 10^9^/L, and for weekly paclitaxel neutrophils ≥1.5 × 10^9^/L and thrombocytes ≥50 × 10^9^/L.

Table 3 shows the estimated risk of febrile neutropenia related to doxorubicin/carboplatin, paclitaxel/carboplatin, capecitabine/5-FU and paclitaxel alone.

The estimates may vary depending on the treatment regimen (dose/m^2^, weekly administration (adm) vs three weekly (q3w), age, comorbidity, treatment intention and performance status [18].

During intermediate or high-risk treatments, added treatment with myeloid growth factor or G-CSF may be necessary. As a growth factor, G-CSF stimulates the genesis of white blood cells. This is especially important during adjuvant treatment in order to maintain dose intensity in case of persisting neutropenia or as a prophylaxis for febrile neutropenia [18]. Side effects are rarely severe and mostly consist of flu-like symptoms [9, 18]. Treatment with G-CSF is rarely used when the patient is being treated with capecitabine since it has been suggested that the combination may increase the myelotoxicity of capecitabine through the proliferative activity of the bone marrow [19].

Ovarian cancer treatment with poly (ADP-ribose) polymerase inhibitors (PARPi), such as olaparib, is approved as maintenance therapy in platinum-sensitive breast cancer gene (BRCA)-mutated relapsed ovarian cancer [20]. Olaparib is administered as capsules consumed twice daily (maximum dosage 400 mg × 2) on a continuous basis. Every treatment cycle is 28 days and requires neutrophils ≥1.5 × 10^9^/L and thrombocytes ≥75 × 10^9^/L [21].

## Case 1

A 57-year-old woman, diagnosed with left-sided colon cancer in 2018 and with synchronous liver metastasis, was referred to the Department of Oncology, Regional Hospital West Jutland, for adjuvant chemotherapy after surgical removal of both primary tumour and liver metastasis.

The woman had no concomitant diseases and took no medication at time of referral.

**Figure 1.**
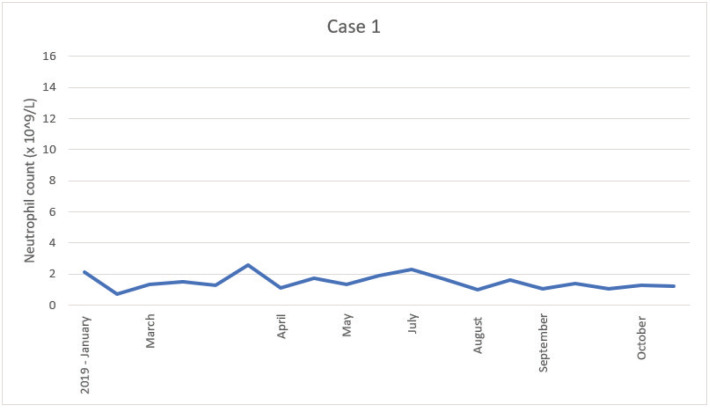
Illustration of the neutrophil count since cancer surgery was performed.

The patient was candidate to adjuvant treatment with capecitabine. However, due to unexplained mild neutropenia, the start of the chemotherapy was postponed several times, fewer treatments were administered and the dose was reduced (Table 4). The patient was never admitted with febrile neutropenia. The patient was referred to the Department of Haematology in November 2019 for diagnostic work-up. Myelodysplastic syndrome (MDS) was excluded as a reason for the neutropenia. Figure 1 shows the level of neutropenia from the date of surgery (November 2018) until treatment was terminated (October 2019).

## Case 2

A 67-year-old woman, diagnosed with FIGO stage IIIC BRCA-mutated high-grade serous ovarian adenocarcinoma in 2016, underwent macroscopic radical surgery, including hyperthermic intraperitoneal chemotherapy. She was referred to the Department of Oncology, Regional Hospital West Jutland, in order to receive adjuvant chemotherapy.

The patient had no concomitant diseases except for allergy-induced asthma that required no daily medication.

Due to chronic moderate neutropenia even before chemotherapy initiation, treatment was postponed several times and the dose was significantly reduced despite addition of G-CSF (Table 5).

**Figure 2.**
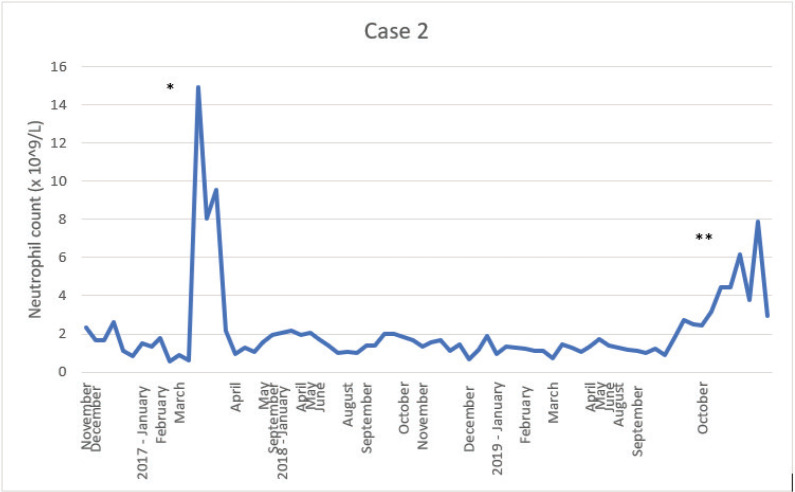
Illustration of the neutrophil count since cancer surgery was performed.

In the palliative setting, the patient became candidate for olaparib treatment; it was started, but stopped after only two cycles due to progression and persistent neutropenia. Later in the treatment course, a weekly paclitaxel regime with reduced requirements to the neutrophil count (>1.0 × 10^9^/L) was administered, but treatments were further postponed and dose-reduced several times despite lower neutrophils were accepted. All along the treatment course, the patient reported general well-being and no episode of fever. Figure 2 shows the level of neutropenia since surgery was performed and up until November 2019 when the patient, due to a decline in her general condition, was treated with corticosteroids daily, and future data were therefore no longer valid.

In September 2018, the patient was referred to the Department of Haematology and a bone marrow biopsy was carried out. MDS was excluded as a reason for the neutropenia. The bone marrow was described as hypoplastic but not dysplastic. Human immunodeficiency virus-testing was negative.

The Department of Pharmacology was contacted in November 2019 to investigate whether the neutropenia could be drug-induced, but no plausible explanation was found. Treatment has since been terminated due to the decline of the patient’s general condition (January 2020).

## Discussion

Optimal treatment with chemotherapy relies entirely on intervals and dose. If dose intensity is compromised, then treatment efficacy is assumed being suboptimal.

If the intervals are prolonged due to neutropenia, then there is a risk of less effective chemotherapy. This phenomenon is demonstrated in the ‘fractional kill’ and ‘log cell kill’ hypothesis [22]. Likewise, if neutropenia results in a lower dosage of chemotherapy, then the risk of insufficient antineoplastic treatment is higher. In line with this, we believe that the treatment with non-cytotoxic drugs is sub-optimal if postponements and dose reductions are implemented due to neutropenia.

Chemotherapy guidelines clearly state that no person should be treated with chemotherapy if the neutrophils are below a predefined level. The guidelines therefore do not take individual patient characteristics into consideration and leave no room for individual medical assessment.

In both cases presented here, the neutropenia was present even before chemotherapy was initiated and gave neither patient any symptoms. The other cell lines remained normal. These patients may have had asymptomatic neutropenia for years. In both cases, it was not possible to maintain dose intensity due to unexplained neutropenia, possibly resulting in less effective treatment. Furthermore, both patients had a markedly delayed initiation of treatment due to neutropenia when they were first referred to the Department of Oncology.

No particular reason for neutropenia was explained in the two clinical cases and many conditions may lead to chronic neutropenia. Cancer, chemotherapy (and other drugs), infections, metabolic disorders or vitamin deficiencies among other factors can cause neutropenia [9].

Conditions such as cyclic neutropenia, AIN and CIN are rare and difficult to diagnose [23].

Cyclic neutropenia is a particularly rare condition in which the neutrophil count fluctuates between abnormal and normal values. Episodes of neutropenia would happen every 2–5 weeks [23]. This is not the case for the two patients here, as the level of neutropenia has not changed over time.

Since the condition in both clinical cases presented here had been present for more than 3 months and other apparent reasons had been excluded, CIN or AIN seems more likely. CIN seems to match both cases in terms of age frequency, gender distribution and chronic tendency. However, AIN cannot be excluded here since none of the cases was tested for autoimmune antibodies. In AIN, neutrophils rise after steroid treatment due to a reduction in the destruction and a glucocorticoid-induced demargination, which is consistent with case #2 (Figure 2).

Different suggestions in terms of treatment of the neutropenia can be speculated but requires thorough investigation with regard to the cause. Treatment could consist of immunoglobulin or methotrexate depending on the underlying reason for the condition. However, these treatments are usually only recommended when the patient have symptoms due to the neutropenia, which was not the case here. The treatment combination of chemotherapy and methotrexate can also be an issue.

CIN/AIN is the most plausible explanation to the neutropenia described in the above-mentioned cases. The objective of this article, however, is not to diagnose the underlying condition that caused the neutropenia in the first place, but to illustrate the complexity of dealing with such patients within the framework of the current guidelines.

The question that remains is whether asymptomatic patients with unexplained neutropenia (due to CIN or AIN) should be treated under the same restrictions as other patients or whether it would be appropriate for an individual clinical judgment to deviate from regulations in cases such as these to avoid insufficient antineoplastic treatment.

## Conclusion

In conclusion, dose intensity is crucial for many antineoplastic treatments, and current guidelines leave no room for personalised treatment of cancer patients with chronic neutropenia. Furthermore, when patients present with unexplained neutropenia before cancer treatment initiation, a haematological examination should be carried out quickly to avoid unnecessary postponement of the cancer treatment initiation. We still know little about how to treat chronic neutropenia in cancer patients, and whether it should be treated at all.

## Disclosures

Niels Fristrup MD PhD, Advisory Board Member, Pfizer inc.

GL: No relevant COIs

Beyond this, authors declare that no financial support or other benefits from commercial sources have been received and none of the authors has any financial or otherwise competing interests that could create any conflict of interests.

## Funding

The authors received no financial support for the research, authorship, and/or publication of this article.

## Figures and Tables

*Increase in neutrophil count to 14.92 × 109/L due to one treatment with G-CSF (Longuex 6 mg) –no chemotherapy was given. **Increase in neutrophil count due to hospital admittance and treatment with corticosteroids.

**Table 1.** Anti-cancer drugs leading to neutropenia.

Drug type	Examples
**Targeted agents**	
Monoclonal antibodies	Rituximab (CD20), daratumumab (CD38), infliximab (TNF), venetoclax (BCL2)
Cyclin-dependent kinase 4/6 inhibitors	Palbociclib, ribociclib, abemaciclib
Protein kinase inhibitors	Ibrutinib
PARP inhibitors	Olaparib, niraparib, rucaparib, veliparib
**Chemotherapy**	
Antimetabolites	5-flourouracil, methotrexate, capecitabine, gemcitabine
Alkylating agents	Cyclophosphamide, carboplatin, oxaliplatin, cisplatin, lomustine
Anti-tumour antibiotics	Doxorubicin, epirubicin, mitomycin C
Topoisomerase inhibitors	Irinotecan, topotecan, etoposide
Plant alkaloids	Vinorelbine, paclitaxel, docetaxel, etoposide, irinotecan, topotecan

**Table 2.** Haematological examination of potential conditions/causes of chronic neutropenia.

Current symptoms and history (including use of drugs, malnutrition, comorbidity, recent travels or primary haematological malignancy)
Concomitant symptoms
Specific blood test to exclude rheumatological or haematological conditions
Demographic factors
Social details
Past medical history (ethnicity, frequent hospitalisation as a child, recent infections, etc.)

**Table 3.** Risk of febrile neutropenia varies among anti-cancer drugs.

Regimen	Estimated risk of febrile neutropenia	References
Doxorubicin/carboplatin	Low (≤10%)	[17, 18]
Paclitaxel/carboplatin	Low (≤10%)	[17, 18]
Capecitabine/5-FU	Low to intermediate (<20%)	[17, 18]
Paclitaxel alone	High (>20%)	[17, 18]

**Table 4.** Treatment overview case #1.

Intended treatment	Capecitabine (1,000 mg/m^2^ twice daily) 1,500 mg (100%) day 1–14 q3w, 8 series in total
Final treatment	Capecitabine 1,150 mg (75%) day 1–14, 6 series in total
Postponements due to low neutrophil count	11 times in total (each lasting 1 week)

**Table 5.** Treatment overview #2.

Intended treatment	Paclitaxel 287 mg (100%) and carboplatin 640 mg (AUC 5) q3w, six series in total
Planned treatment after relapse	Carboplatin 609 mg (AUC 5) + G-CSF (Longuex 6 mg) q3w, 3–6 series in total, and then treatment with Olaparib 400 mg × 2 (100%) q4w^*^
Administered treatment	Olaparib terminated after only two series. Hereafter: Paclitaxel 77 mg (60%) weekly administered
Postponements due to low neutrophil count	16 times in total (each lasting 1 week)

^*^ At this time, only Olaparib capsules were available
